# High mobility group box-1 contributes to anti-myeloperoxidase antibody-induced glomerular endothelial cell injury through a moesin-dependent route

**DOI:** 10.1186/s13075-017-1339-4

**Published:** 2017-06-06

**Authors:** Hui Deng, Chen Wang, Dong-Yuan Chang, Nan Hu, Min Chen, Ming-Hui Zhao

**Affiliations:** Renal Division, Department of Medicine, Peking University First Hospital, Institute of Nephrology, Peking University; Key Laboratory of Renal Disease, Ministry of Health of China, Beijing, 100034 China

**Keywords:** HMGB1, Myeloperoxidase, Antineutrophil cytoplasmic antibody, Moesin, Glomerular endothelial cell

## Abstract

**Background:**

Our previous study found that circulating and urinary levels of high mobility group box-1 (HMGB1) were closely associated with disease activity in patients with antineutrophil cytoplasmic antibody (ANCA)-associated vasculitis (AAV). Moreover, HMGB1 participates in ANCA-induced neutrophil activation. Cross-reactivity between moesin and anti-myeloperoxidase (MPO) antibody has been reported in both human and mouse. The current study investigated whether HMGB1 participated in MPO-ANCA-induced glomerular endothelial cell (GEnC) injury, which is one of the most important aspects in the pathogenesis of AAV.

**Methods:**

The effects of HMGB1 on expression of moesin on GEnCs and anti-MPO antibody binding to GEnCs were measured. MPO expression on GEnCs was explored. The effects of HMGB1 in MPO-ANCA induced GEnC injury were measured, during which the role of moesin was explored. Antagonists for various relevant receptors were employed.

**Results:**

Sera from AAV patients at the active stage could mediate GEnC injury, while this effect could be attenuated by preblocking HMGB1. HMGB1 could increase the expression of moesin on GEnCs and the binding of anti-MPO antibody to moesin. The colocalization of moesin expression and anti-MPO antibody binding can be detected. Little, if any, MPO was expressed in GEnCs. HMGB1 increased GEnC activation and injury in the presence of patient-derived MPO-ANCA-positive IgGs through moesin. The effects of HMGB1 on expression of moesin on GEnCs, anti-MPO antibody binding to GEnCs, GEnC activation and injury were mainly toll like receptor 4 (TLR4) dependent.

**Conclusions:**

HMGB1 can increase the expression of moesin but not MPO on GEnCs, and can further participate in MPO-ANCA-induced GEnC activation and injury by cross-reactivity between moesin and anti-MPO antibody.

**Electronic supplementary material:**

The online version of this article (doi:10.1186/s13075-017-1339-4) contains supplementary material, which is available to authorized users.

## Background

Antineutrophil cytoplasmic antibody (ANCA)-associated vasculitis (AAV) consists of granulomatosis with polyangiitis (GPA, previously named Wegener’s granulomatosis), microscopic polyangiitis (MPA) and eosinophilic granulomatosis with polyangiitis (EGPA) [1]. The serological markers for the aforementioned primary small vessel vasculitis are ANCAs, which recognize a variety of target antigens, in particular proteinase 3 (PR3) and myeloperoxidase (MPO). It is worth noting that Chinese patients with AAV are predominantly MPO-ANCA-positive, as demonstrated by our previous studies [2, 3]. One of the hallmarks of AAV is massive endothelial injury, especially glomerular endothelial cell (GEnC) injury, resulting in necrotizing vasculitis. ANCAs are proved to be involved in inducing and amplifying neutrophil-mediated endothelial injury in AAV [4, 5]. Nevertheless, there is also evidence supporting the direct ability of MPO-ANCA to produce vessel damage [6–8].

High mobility group box-1 (HMGB1) exists ubiquitously within the nucleus, playing its role of stabilizing the structure of nucleosomes and inducing DNA bending [9]. Upon certain stimulations, HMGB1 is released from various cells and becomes a proinflammatory mediator [10]. The signal pathways of HMGB1 involve a number of signaling molecules and receptors, including receptor for advanced glycation end products (RAGE) and Toll-like receptors TLR2 and TLR4 [11–13]. In our recent studies, we found that circulating and urinary levels of HMGB1 are associated with disease activity and renal damage in AAV patients [14, 15]. Moreover, HMGB1 participates in ANCA-induced neutrophil activation, indicating a pathogenic role of HMGB1 in AAV [16, 17].

Recently, Lee et al. [18] demonstrated that the HMGB1–RAGE–moesin axis could elicit severe inflammatory responses on human umbilical vein endothelial cells (HUVEC), during which HMGB1 exhibited an increase in phosphorylation of moesin and further secretion of moesin. Moesin, with the full name of membrane-organizing extension spike protein, is previously described as a cytoskeletal protein that belongs to the ezrin–radixin–moesin (ERM) family, which could function as links between the plasma membrane and the actin cytoskeleton [19]. More interestingly, Nagao et al. recently reported a direct activation of mouse GEnCs by anti-moesin activity of anti-MPO antibody [8]. In their study, the authors identified a cross-reactive molecule, which could be recognized by anti-MPO antibody, existing on mouse GEnCs. Later, the molecule was confirmed as moesin by mass spectrometry [8]. Given the potential effect of HMGB1 on upgrading moesin and the anti-moesin activity of anti-MPO antibody on GEnCs, we hypothesized that there is a moesin-dependent way through which HMGB1 can contribute to MPO-ANCA-induced GEnC injury.

## Methods

### Reagents

Recombinant HMGB1 protein was purchased from R&D Systems (C23-C45 disulfide C106 thiol form; Abingdon, UK). The endotoxin level of HMGB1 was below the detection limit (0.125 EU/ml) of the Limulus assay (Sigma, St Louis, MO, USA). Anti-HMGB1 IgY was purchased from Shino-TEST (Sagamihara, Japan). Tumor necrosis factor alpha (TNF-α), lipopolysaccharides (LPS) and polymyxin B were purchased from Sigma. Monoclonal antibodies (mAbs) recognizing human moesin and MPO were purchased from Abcam (Cambridge, UK), with irrelevant IgG control antibodies. Polyclonal antibodies to MPO were purchased from Millipore (CA, USA). For inhibition assay, antibodies blocking moesin were purchased from BD Biosciences (San Jose, CA, USA). Normal human IgG and rabbit IgG were purchased from Sigma. Antibodies blocking the TLR2 and TLR4 were purchased from eBioscience (San Diego, CA, USA). RAGE-Fc was purchased from R&D Systems (Minneapolis, MN, USA). For immunofluorescence assay, AF488-labeled donkey anti-rabbit IgG and Cy3-labeled donkey anti-mouse IgG were purchased from Jackson ImmunoResearch Laboratories (Westgrove, PA, USA). For enzyme-linked immunosorbent assay (ELISA) experiments, human moesin protein was purchased from Sino Biological Inc. (Beijing, China). For flow cytometry analysis, phycoerythrin (PE)-conjugated goat anti-rabbit antibody and allophycocyanin (APC)-conjugated goat anti-mouse antibody were purchased from Abcam. For immunoprecipitation assay, rabbit anti-MPO antibody was purchased from BD Pharmingen (San Jose, CA, USA), while the control rabbit IgG was purchased from Santa Cruz (CA, USA). Peroxidase conjugated donkey anti-mouse IgG was purchased from Santa Cruz.

### Patients

Blood samples of 10 active AAV patients with positive MPO-ANCA at initial onset and before commencing immunosuppressive therapy, diagnosed at Peking University First Hospital from August 2013 to June 2014, were collected in this study. All of the patients met the Chapel Hill Consensus Conference (CHCC) definition of AAV [1]. Patients with secondary vasculitis or with comorbid renal diseases, such as anti-glomerular basement membrane (GBM) nephritis, IgA nephropathy, diabetic nephropathy, lupus nephritis or membranous nephropathy, were excluded. Blood samples of five age and sex-matched healthy controls were collected. Venous blood was collected into red cap vacuum blood collection tubes. Collections of the whole blood sample were centrifuged at 3000 rpm for 10 min at 4 °C. Sera were then obtained and kept at –80 °C until use. The general information for these patients and healthy controls is presented in Table 1.

**Table 1:** General data for AAV patients and controls

Characteristic	AAV (*n* = 10)	HC (*n* = 5)
Sex (male/female)	6/4	3/2
Age (years), median (range)	60.5 (39–72)	61.5 (40–65)
Scr (μmol/L), mean ± SD	620.8 ± 320.0	–
ESR (mm/h), mean ± SD	70.4 ± 36.3	–
BVAS, median (range)	21.5 (15–32)	–
ANCA (RU/ml)	All > 200	Negative
Organ involvement, *n* (%)		
ENT	3 (30%)	–
Lung	7 (70%)	–
Kidney	10 (100%)	–
Skin	2 (20%)	–

*AAV* ANCA-associated vasculitis, *ANCA* antineutrophil cytoplasmic antibody, *BVAS* Birmingham Vasculitis Activity Score, *ENT* ear, nose and throat, *HC* healthy control, *Scr* serum creatinine, *ESR* erythrocyte sedimentation rate, *RU﻿ * relative unit

### Preparation of IgG

MPO-ANCA-positive IgGs were prepared from plasma exchange liquid of patients with active MPO-ANCA-positive primary small vessel vasculitis, using a High-Trap-protein G column on an AKTA-FPLC system (GE Biosciences, South San Francisco, CA, USA). Preparation of IgG was performed according to the methods described previously [20, 21]. In brief, plasma exchange liquid was filtered through a 0.2-mm syringe filter (Schleicher & Schuell, Duesseldorf, Germany) and applied to a High-Trap-protein G column on an AKTA-FPLC system (GE Biosciences). The column was treated with equal volume of 20 mmol/L Tris–HCl buffer, pH 7.2 (binding buffer), and IgG was eluted with 0.1 mol/L glycine–HCl buffer, pH 2.7 (elution buffer). After the antibodies emerged from the column, the pH was immediately adjusted to pH 7.0 using 2 mol/L Tris–HCl (pH 9.0). The protein concentration of the antibodies was measured using the Nandrop-1000 (Pierce, Rockford, IL, USA), and the level of anti-MPO IgG was measured by the ELISA kit (EUROIMMUN, Lubeck, Germany). We obtained written informed consent from the participants involved in our study. The research was in compliance of the Declaration of Helsinki and approved by the clinical research ethics committee of the Peking University First Hospital.

### Cell culture

Primary GEnCs (ScienCell, San Diego, CA, USA) were cultured in endothelial cell basal medium (ECM) (ScienCell) with additional 10% fetal bovine serum (FBS), 1% penicillin/streptomycin and 1% endothelial cell growth factor in the formation of a confluent endothelial cell monolayer. The flasks for cell subculture were biocoated with human plasma fibronectin (Millipore, Billerica, MA, USA) beforehand according to the manufacturer’s recommendation. For synchronization of the cell cycle, GEnC monolayers were starved in basal medium without FBS and endothelial cell growth supplement for 12 h without biocoating. All experiments were performed using GEnCs at passages 3–5. All cultures were incubated at 37 °C in 5% CO_2_. In order to investigate the effect of HMGB1 in the sera on GEnC injury, GEnC monolayers were incubated with ECM with additional 10% sera from either AAV patients or healthy controls for 4 h at 37 °C. For HMGB1 inhibition, GEnC monolayers were preincubated with 10 μg/ml anti-HMGB1 IgY for 1 h, which is the commercial anti-HMGB1 blocking antibodies isolated and purified from the egg yolk of HMGB1-immunized hens, followed by other treatments.

### Measurement of moesin expression and the binding of anti-MPO mAb on GEnCs

#### Flow cytometry

The GEnC monolayers were incubated for 4 h with HMGB1 at a concentration of 10 ng/ml, which was comparable with the circulating HMGB1 level in active AAV patients [15], TNF-α, LPS, polymyxin B or buffer control. The time was set according to the result of time-dependent curve and cell conditions. In order to further investigate the role of candidate receptors of HMGB1 on GEnCs, the cells were first incubated with blocking antibodies and inhibitors (anti-TLR2 at 5 μg/ml; anti-TLR4 at 5 μg/ml; RAGE-Fc at 5 μM) or buffer control for 2 h. Next, cells were digested using trypsin to maintain in suspension. After washing, suspended cells were incubated with Trustain FcX™ Fc receptor blocking solution (Biolegend, CA, USA) for 10 min, and then stained with a saturating dose of rabbit antibody against human moesin or with an irrelevant IgG control antibody for 30 min, followed by incubation with PE-conjugated goat anti-rabbit antibody for another 30 min. For double staining of moesin and MPO on GEnCs, the suspended cells were stained with saturating doses of rabbit antibody against human moesin and mouse antibody against human MPO, or with irrelevant IgG control antibodies for 30 min, followed by incubated with both PE-conjugated goat anti-rabbit antibody and APC-conjugated goat anti-mouse antibody for 30 min consistently. After washing with phosphate buffer saline (PBS), prepared cells were analyzed using a FACSCalibur flow cytometer (Influx™). Cells were gated in forward/sideward scatter (FSC/SSC) and data were collected from 10,000 cells per sample.

#### Immunofluorescence

For fluorescent microscopy, GEnCs were grown for 48–72 h in slide culture chambers (Nunc, Roskilde, Denmark) until confluent. The procedures were conducted as described previously [22]. After treatment, the GEnC monolayers were fixed in cold 4% paraformaldehyde (PFA) for 4 h at 4 °C. After multiple washing with PBS, coverslips with the fixed cells were removed from the plates. Nonspecific binding sites were blocked in PBS containing 5% donkey serum. We incubated the specimens with rabbit mAb against moesin (1:100 dilution) combined with mouse antibody against MPO (1:100 dilution) in blocking buffer for 1 h at 37 °C. After washing with PBS, AF488-labeled donkey anti-rabbit IgG or Cy3-labeled donkey anti-mouse IgG (both 1:500 dilution) was applied for 1 h. The samples were washed with PBS and distilled water, stained with DAPI and mounted with Mowiol. All images were collected and analyzed with a fluorescent microscope (Nikon 80I). The percentage of anti-MPO antibody binding with moesin was analyzed with Image-Pro Plus v6.0.

#### Mass spectrometry

GEnCs were cultured on a culture plate until confluence. After washing twice with PBS, the cells were lysed and the sample was separated by SDS-PAGE. After identification by coomassie blue staining, the bands on gel were divided into 10 parts according to protein abundance and molecular weights. Each part of the sample was applied to mass spectrometry to detect the level of MPO expression in GEnCs, in order to explore the possibility of anti-MPO antibody binding with intrinsic MPO in GEnCs.

#### Immunoprecipitation

After treatment, the GEnC monolayers were lysed in ice-cold RIPA buffer at 4 °C for 30 min. The cell lysates were then mixed with polyclonal antibodies to MPO, or rabbit normal IgG as control, and incubated overnight at 4 °C on a rotating device. After mixing with Protein A/G PLUS-Agarose (Santa Cruz) for another 3 h at 4 °C, the immunoprecipitates were washed in RIPA buffer five times, and then the boiled samples were separated by SDS-PAGE and detected by anti-moesin mAb. Finally, the strips were incubated with peroxidase-conjugated affinity-purified donkey anti-mouse IgG (1:5000 dilution) for 1 h at room temperature with gentle agitation and then were revealed on autoradiographic film using the ECL Plus Western Blotting Detection System (GE Healthcare, Piscataway, NJ, USA).

#### ELISA

The binding of moesin and anti-MPO antibody was further detected by ELISA with human moesin at 2 μg/ml as the solid-phase antigen, bovine serum albumin (BSA) at the same concentration as control and anti-MPO polyclonal antibody (1:2000 dilution) as the detection antibody. Anti-MPO antibody was added to the antigen-coated microtiter plate and incubated for 1 h at 37 °C. Anti-rabbit IgG alkaline phosphatase antibody produced in goat was then added to the wells. After adding to the stop solution, the absorption measurement was obtained at 405 nm using a microtiter plate reader (Bio-Rad iMark™ Microplate Reader).

### Measurements of GEnC activation and injury in vitro

#### Measurement of soluble ICAM-1 and soluble VCAM-1

Levels of soluble intercellular cell adhesion molecule-1 (sICAM-1) and soluble vascular cell adhesion molecule-1 (sVCAM-1) were considered biomarkers of endothelial cell activation and injury [23]. After treatment, the cell culture supernatant was collected for the ICAM-1 and VCAM-1 assays. Samples were tested using the human ICAM-1/CD54 and human VCAM-1/CD106 ELISA kit (R&D, Abingdon, UK). The assay was conducted according to the manufacturer’s instructions. In brief, samples were added to the microtiter plate coated with capture antibody and incubated for 2 h at room temperature, followed by detection antibody incubation for another 2 h. Horseradish peroxidase-conjugated streptavidin was then added. After 20 min of incubation avoiding direction light, the plates were washed and substrate solution was added to the wells. After adding to the stop solution, the absorption measurements were obtained at 450 nm (with a correction of 570 nm to eliminate optical imperfections in the plate) using a microtiter plate reader (Bio-Rad iMark™ Microplate Reader). All samples and standards were performed in duplicate.

#### Cytotoxicity assay

The cytotoxic effect of HMGB1 or/and MPO-ANCA-positive IgGs was determined by measuring the secretion of lactate dehydrogenase (LDH) using the Cytotoxicity Detection Kit (Roche Diagnostics, Mannheim, Germany) according to manufacturer’s recommendation. To demonstrate the effects of moesin in this process, the cells were preincubated with blocking antibodies to moesin with a saturated dosage before treated with HMGB1 and/or MPO-ANCA-positive IgGs.

#### Permeability assay

GEnC monolayer permeability was determined with fluorescein isothiocyanate (FITC)-labeled BSA (Sigma), as described previously [24]. Cells were grown on Costar Transwell 0.4-μm porous filters (Coming, Acton, USA) until confluent. Also, in order to demonstrate the effect of moesin in this process, the cells were preincubated with blocking antibody to moesin before treatment with anti-MPO antibody, MPO-ANCA-positive IgGs and/or HMGB1. After treatment, the tracer protein FITC-albumin was added to the luminal chamber for 45 min, and samples were taken from both the luminal and abluminal chambers for fluorometry analysis. Fluorescence signals were measured in a microplate fluorescence reader (Tristar™ LB941) with filter settings of 485 nm (excitation) and 538 nm (emission). These readings were then used to determine the permeability coefficient of albumin which could stand for the vascular barrier disruption. All data are shown as the ratio of control at the corresponding test.

### Statistical analysis

Quantitative data were expressed as mean ± SD (for data that were normally distributed) or median and quartiles (for data that were not normally distributed) as appropriate. Differences of quantitative parameters between groups were assessed using one-way ANOVA (for data that were normally distributed) or Mann–Whitney *U* test (for data that were not normally distributed) as appropriate. Differences were considered significant when *p* < 0.05. Analysis was performed with SPSS statistical software package (version 13.0; Chicago, IL, USA).

## Results

### HMGB1 participated in GEnC injury mediated by sera from AAV patients

**Fig. 1:**
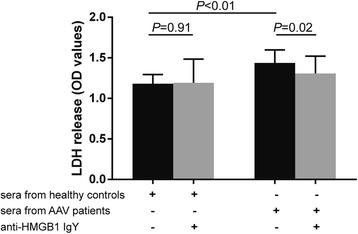
HMGB1 participated in the GEnC injury mediated by sera from AAV patients. Levels of LDH release increased significantly in GEnCs incubated with ECM containing sera from AAV patients and decreased significantly by preincubating anti-HMGB1 IgY. *Bars* represent mean ± SD of repeated measurements from three independent experiments. *AAV* ANCA-associated vasculitis, *ANCA* antineutrophil cytoplasmic antibody, *HMGB1* high mobility group box-1, *LDH* lactate dehydrogenase, *OD* optical density

Compared with GEnCs incubated with ECM with additional 10% sera from healthy controls, the levels of LDH release increased significantly in GEnCs incubated with ECM with additional 10% sera from AAV patients (1.44 ± 0.16 vs 1.18 ± 0.11, *p* < 0.01). By preincubating anti-HMGB1 IgY, the levels of LDH release decreased significantly in GEnCs incubated with ECM with additional 10% sera from AAV patients (1.44 ± 0.16 vs 1.30 ± 0.21, *p* = 0.02) (Fig. 1).

### HMGB1 increased the expression of moesin on GEnCs

**Fig. 2:**
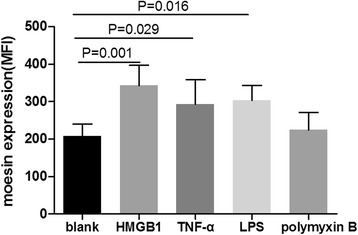
HMGB1 increased the expression of moesin on GEnCs. Expression of moesin on GEnCs was significantly increased with the treatment of HMGB1, LPS and TNF-α. *Bars* represent mean ± SD of repeated measurements from three independent experiments. *HMGB1* high mobility group box-1, *LPS* lipopolysaccharides, *MFI* mean fluorescence intensity, TNF-α, Tumor necrosis factor alpha

Expression of moesin on GEnCs in different experimental groups in vitro was analyzed using flow cytometry. Compared with untreated cells, the level of moesin expression was significantly higher on GEnCs treated with HMGB1 at concentration of 10 ng/ml, TNF-α at 15 ng/ml or LPS at 90 ng/ml (343 ± 55 vs 207 ± 33, *p* < 0.01; 293 ± 66 vs 207 ± 33, *p =* 0.03; or 303 ± 41 vs 207 ± 33, *p =* 0.02, respectively). No obvious moesin expression increase was observed in cells treated with polymyxin B at 20 μg/ml (224 ± 47 vs 207 ± 33, *p* = 0.65) (Fig. 2).

### Binding of anti-MPO mAb to GEnCs treated with HMGB1 through moesin increased significantly

Given the cross-reaction to moesin of anti-MPO antibody [8], we studied whether the binding of anti-MPO mAb to GEnCs increased with the treatment of HMGB1.

**Fig. 3:**
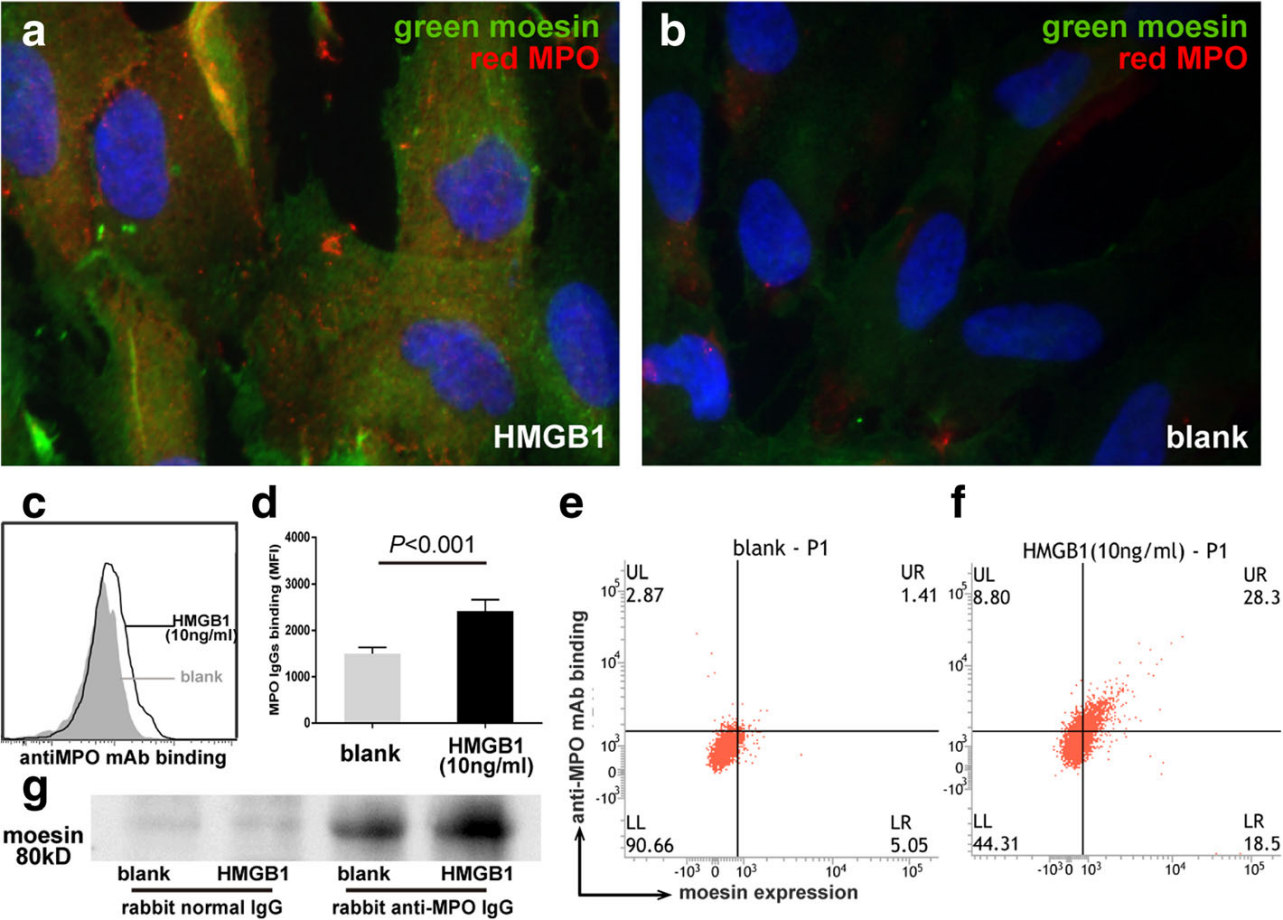
HMGB1 increased the binding of anti-MPO mAb to GEnCs. Compared with untreated cells, moesin expression and the binding of anti-MPO monoclonal antibody (*mAb*) were upregulated on HMGB1-treated GEnCs (**a**, **b**). Binding of anti-MPO mAb significantly increased on HMGB1-treated GEnCs compared with untreated cells (**c**). *Bars* represent mean ± SD of repeated measurements from three independent experiments. Representative histogram of effects of HMGB1 on the binding of anti-MPO monoclonal antibody to GEnCs (**d**). Proportion of GEnCs with both upregulated moesin expression and increased anti-MPO monoclonal antibody binding elevated in HMGB1-treated cells (**e**, **f**). Moesin on GEnCs could be captured by polyclonal antibodies to MPO by immunoprecipitation (**g**). *HMGB1* high mobility group box-1, *MPO* myeloperoxidase, *MFI* mean fluorescence intensity

Indirect immunofluorescent assay was performed to study the expression of moesin and the binding of anti-MPO mAb to GEnCs. Compared with untreated cells, moesin expression and the binding of anti-MPO mAb significantly increased on HMGB1-treated GEnCs. Moreover, colocalization of moesin expression and anti-MPO mAb binding were more obvious in HMGB1-treated cells than in untreated cells (Fig. 3a, b). The percentage of colocalization for moesin and anti-MPO mAb was about 6% after HMGB1 stimulation.

Mass spectrometry showed that little, if any, MPO was expressed in GEnCs, suggesting that the binding of anti-MPO antibody with moesin detected by immunofluorescent assay was not via MPO (see Additional file 1: Table S1 and Additional file 2: Table S2).

Flow cytometry assay was further performed to study the change of proportions of GEnCs with both upregulated moesin expression and increased anti-MPO mAb binding. Compared with untreated cells, the binding of anti-MPO mAb increased significantly (2418 ± 248 vs 1497 ± 139, *p* < 0.01), and the proportion of GEnCs with both upregulated moesin expression and increased anti-MPO mAb binding elevated in HMGB1-treated cells also increased (Fig. 3c–f).

The binding of anti-MPO IgG to moesin was confirmed by immunoprecipitation. It was found that moesin in GEnCs was recognized by anti-MPO antibody (Fig. 3g). The binding was further detected by ELISA, showing that the level of anti-MPO antibody binding to coated moesin was significantly higher than that of anti-MPO antibody binding to BSA control (expressed by OD values: 0.03 ± 0.0040 vs –0.016 ± 0.011, *p* < 0.01).

### HMGB1 increased GEnC activation and injury in the presence of patient-derived MPO-ANCA-positive IgGs, while HMGB1-induced GEnC injury was attenuated by blocking moesin

We next studied the effect of HMGB1 on activation and subsequent injury of GEnCs.

**Fig. 4:**
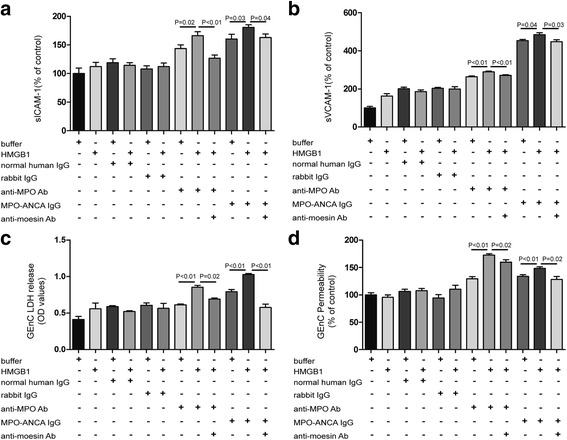
HMGB1 increased GEnC activation and injury in the presence of patient-derived MPO-ANCA-positive IgGs through moesin. Levels of both sICAM-1 and sVCAM-1 increased significantly in the supernatants of GEnCs treated with HMGB1 plus patient-derived MPO-ANCA-positive IgGs compared with untreated cells, cells treated with HMGB1 or patient-derived MPO-ANCA-positive IgGs alone (**a**, **b**). Levels of LDH release increased significantly in the supernatants of GEnCs treated with HMGB1 plus patient-derived MPO-ANCA-positive IgGs compared with untreated cells, cells treated with HMGB1 or patient-derived MPO-ANCA-positive IgGs alone (**c**). Levels of vascular barrier disruption increased significantly in GEnCs treated with HMGB1 plus patient-derived MPO-ANCA-positive IgGs compared with untreated cells, cells treated with HMGB1 or patient-derived MPO-ANCA-positive IgGs alone (**d**). By preincubating with anti-moesin antibodies, levels of cell activation, LDH release and levels of vascular barrier disruption decreased significantly (**a**–**d**). Bars represent mean ± SD of repeated measurements from five independent experiments. *Ab* antibody, *ANCA* antineutrophil cytoplasmic antibody, *GEnC* glomerular endothelial cell, *HMGB1* high mobility group box-1, *LDH* lactate dehydrogenase, *MPO* myeloperoxidase, *OD* optical density, *sICAM*-1, soluble intercellular cell adhesion molecule-1, *sVCAM*-1, soluble vascular cell adhesion molecule-1

sICAM-1 and sVCAM-1 were considered markers of endothelial cell activation as already mentioned. Compared with untreated cells, the levels of both sICAM-1 and sVCAM-1 increased significantly in the supernatants of GEnCs treated with HMGB1 plus patient-derived MPO-ANCA-positive IgGs (expressed as percentages of the control: 180 ± 19% vs 100%, *p* < 0.01; and 484 ± 29% vs 100%, *p* < 0.01, respectively). Levels of these markers also increased in the supernatants of GEnCs treated with patient-derived MPO-ANCA-positive IgGs alone (160 ± 32% vs 100%, *p* < 0.01; and 453 ± 16% vs 100%, *p* < 0.01, respectively). However, compared with untreated cells, sICAM-1 and sVCAM-1 levels still increased significantly in the supernatants of GEnCs treated with HMGB1 plus anti-MPO polyclonal antibody (166 ± 27% vs 100%, *p* < 0.01; and 289 ± 10% vs 100%, *p* < 0.01, respectively). No obvious GEnC activation was observed in cells treated with normal human IgG, rabbit IgG or HMGB1 alone (Fig. 4a, b).

LDH assay was performed to study the viability of GEnCs in different experimental groups. Compared with untreated cells, cells treated with HMGB1 or patient-derived MPO-ANCA-positive IgGs alone, the levels of LDH release increased significantly in the supernatants of GEnCs treated with HMGB1 plus patient-derived MPO-ANCA-positive IgGs (expressed by OD values: 1.03 ± 0.03 vs 0.41 ± 0.10, *p* < 0.01; 1.03 ± 0.03 vs 0.56 ± 0.20, *p* < 0.01; and 1.03 ± 0.03 vs 0.79 ± 0.06, *p* < 0.01, respectively). Similarly, compared with cells treated with HMGB1 or anti-MPO polyclonal antibody alone, the levels of LDH release increased significantly in cells treated with HMGB1 plus anti-MPO polyclonal antibody (expressed by OD values: 0.85 ± 0.04 vs 0.56 ± 0.19, *p* < 0.05; and 0.85 ± 0.04 vs 0.61 ± 0.02, *p* < 0.01, respectively). No obvious LDH release was observed in cells treated with normal human IgG, rabbit IgG or HMGB1 alone (Fig. 4c).

Endothelial cell permeability assay was performed to study the vascular barrier disruption in different experimental groups. Compared with untreated cells, cells treated with HMGB1 or patient-derived MPO-ANCA-positive IgGs alone, the levels of vascular barrier disruption increased significantly in GEnCs treated with HMGB1 plus patient-derived MPO-ANCA-positive IgGs (expressed as percentages of the control: 148 ± 8.2% vs 100%, *p* < 0.01; 148 ± 8.2% vs 96 ± 14%, *p* < 0.01; and 148 ± 8.2% vs 134 ± 8.9%, *p* < 0.01, respectively). Similarly, significant increase of vascular barrier disruption was also detected in GEnCs treated with HMGB1 plus anti-MPO polyclonal antibody, as compared with cells treated with anti-MPO polyclonal antibody alone (173 ± 6.5% vs 129 ± 15%, *p* < 0.01) (Fig. 4d). By preincubating with anti-moesin antibodies, the levels of sICAM-1 and sVCAM-1, LDH release and the levels of vascular barrier disruption in cells treated with MPO-ANCA-positive IgGs plus HMGB1 decreased significantly (163 ± 28% vs 181 ± 19%, *p* < 0.05; 448 ± 26% vs 484 ± 29%, *p* < 0.05; 0.58 ± 0.12 vs 1.03 ± 0.03, *p* < 0.01; and 128 ± 22% vs 148 ± 8.2%, *p* = 0.02, respectively) (Fig. 4a–d).

Collectively, these data suggested that HMGB1 increased GEnC activation and injury in the presence of patient-derived MPO-ANCA-positive IgGs via a moesin-dependent route.

### Effects of HMGB1 on expression of moesin on GEnCs, anti-MPO mAb binding to GEnCs, GEnC activation and injury were mainly TLR4 dependent

To further investigate the role of candidate receptors through which HMGB1 exerts its effect, GEnCs were preincubated with anti-TLR2 at 10 μg/ml, anti-TLR4 at 10 μg/ml or RAGE-Fc at 10 μM to block corresponsive receptors.

**Fig. 5:**
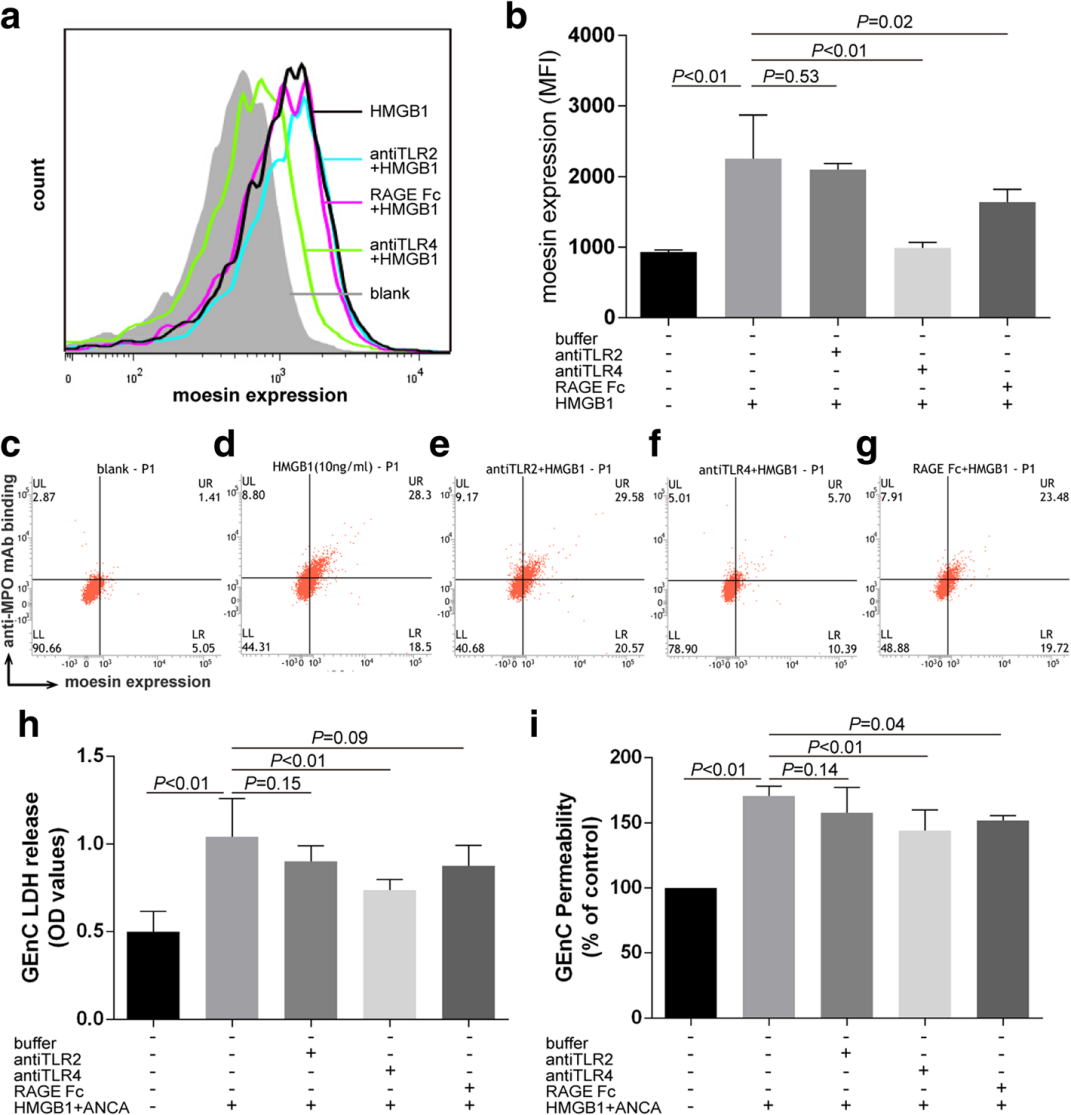
Effects of HMGB1 on expression of moesin on GEnCs, anti-MPO mAb binding to GEnCs, GEnC activation and injury were mainly TLR4 dependent. Representative histogram for expression of moesin on GEnCs (**a**). Blockage of TLR4 and RAGE rather than TLR2 decreased expression of moesin on HMGB1-treated GEnCs (**b**). Among them, TLR4 had a dominate effect. *Bars* represent mean ± SD of repeated measurements on neutrophils for five independent experiments. Blockage of TLR4 rather than RAGE or TLR2 decreased the binding of anti-MPO mAb to GEnCs (**c**–**g**). Blockage of TLR4 rather than RAGE or TLR2 decreased the levels of LDH release (**h**). Blockage of TLR4 and RAGE rather than TLR2 decreased the levels of vascular barrier disruption decreased significantly (**i**). *Bars* represent mean ± SD of repeated measurements on neutrophils from five independent experiments. *mAb* monoclonal antibody, *ANCA* antineutrophil cytoplasmic antibody, *GEnC* glomerular endothelial cell, *HMGB1* high mobility group box-1, *LDH* lactate dehydrogenase, *MPO* myeloperoxidase, *MFI* mean fluorescence intensity, *OD* optical density, *RAGE* receptor for advanced glycation end product, *TLR* Toll-like receptor

Using flow cytometry, parallel experiments blocking TLR4 and RAGE resulted in a significant decrease in HMGB1-induced expression of moesin. Moesin expression decreased from 2258 ± 618 in HMGB1-treated GEnCs, to 991 ± 79 by preincubating with anti-TLR4 antibody (*p* < 0.01) or to 1641 ± 179 by preincubating with RAGE antagonist (*p* = 0.02), while there was no significant decrease in cells preincubated with anti-TLR2 antibody (2258 ± 618 vs 2102 ± 86, *p* = 0.53) (Fig. 5a, b). Subsequently, anti-MPO mAb binding decreased on preincubation with anti-TLR4 antibody and RAGE antagonists, while no significant decrease occurred in GEnCs preincubated with anti-TLR2 antibody (Fig. 5c–g). Although blocking RAGE resulted in a decrease in HMGB1-induced expression of moesin and increased binding of anti-MPO mAb, the change seemed to be mild.

Consistently, the injury induced by HMGB1 and patient-derived MPO-ANCA-positive IgGs also decreased in GEnCs preincubated with anti-TLR4 antibody or RAGE antagonist. In LDH assay, the levels of LDH release decreased significantly in the supernatants of GEnCs treated with HMGB1 plus patient-derived MPO-ANCA-positive IgGs by preincubating with anti-TLR4 antibody (expressed by OD values: 0.74 ± 0.06 vs 1.04 ± 0.22, *p* < 0.01) (Fig. 5h). In endothelial cell permeability assay, the levels of vascular barrier disruption decreased significantly in GEnCs treated with HMGB1 plus patient-derived MPO-ANCA-positive IgGs by preincubating with anti-TLR4 antibody and preincubating with RAGE antagonist (expressed as percentages of the control: 144 ± 16% vs 171 ± 7%, *p* < 0.01; and 152 ± 4% vs 171 ± 7%, *p* = 0.04, respectively) (Fig. 5i). The effects caused by preincubating with RAGE antagonist seemed to be mild. Collectively, the effects of HMGB1 on expression of moesin on GEnCs, anti-MPO mAb binding to GEnCs, GEnC activation and injury were mainly TLR4 dependent.

## Discussion

In the current study, we observed that sera from AAV patients at the active stage could mediate GEnC injury, while the effect could be attenuated by preblocking HMGB1, indicating that HMGB1 participated in the GEnC injury in AAV. Then we further found that HMGB1 could increase the expression of moesin on GEnCs and the binding of anti-MPO mAb to GEnCs due to the cross-reaction of anti-MPO antibody to moesin. In such circumstances, GEnCs could be activated and sequentially be damaged.

As reported by Pendergraft et al. [25], MPO is not expressed in endothelial cells. Nagao et al. identified a cross-reactive molecule on mouse GEnCs, which could be recognized by anti-MPO antibody, termed moesin [8]. They assumed that moesin shares certain similar sequences with those on the N-terminal region of the MPO heavy chain. Furthermore, the same group also demonstrated that MPO-ANCA from patients with active AAV had high reactivity to the aforementioned sequences in human [26, 27]. In our study, we detected the binding of anti-MPO antibody to moesin by immunofluorescence. Also, MPO was scarcely expressed in GEnCs, as measured by protein mass spectrometry, suggesting that the binding of anti-MPO antibody to the GEnC surface was not via MPO. In addition, binding of anti-MPO antibody to moesin was further confirmed by immunoprecipitation and ELISA. However, human MPO has much lower homology with human moesin. Other than molecular mimicry with similar sequences [28], the cross-reaction observed in our study might also be attributed to other mechanisms such as spatial distribution of charge or certain intermediators [29–31], which need further investigations to confirm.

In the current study, sICAM-1 and sVCAM-1 were employed as markers of endothelial cell activation. Generally, biomarkers of endothelial cell activation can be divided into soluble markers, among which sICAM-1, sVCAM-1 and VEGF are typical, and cell component markers, including circulating endothelial cells (CECs) and endothelial microparticles (EMPs) [23, 32]. In AAV, the activated endothelial cells would be further damaged mainly in two ways. First, upregulated cell adhesion molecules make endothelial cells more vulnerable to the attack of neutrophils [4, 33]. Second, the activated cells would then detach and undergo necrosis and apoptosis, thus increasing vascular permeability and causing vascular barrier disruption in specific stimulating conditions [34].

Among the various receptors which HMGB1 can recognize on the cell surface of GEnCs, we found TLR4 is the dominant receptor through which HMGB1 exerts the aforementioned moesin-dependent effects. Lee et al.’s study [18] found that the HMGB1–RAGE–moesin axis could elicit severe inflammatory responses on HUVEC. Different types of endothelial cells and dosages of HMGB1 employed might contribute to such discrepancy of receptors. To be specific, different types of endothelial cells might have different responses to inflammatory conditions or mediators [35, 36], which is also indicates necessity to start with the main affected endothelial cell types involved in the pathogenesis of AAV. We applied HMGB1 at a concentration of 10 ng/ml, which was comparable with the circulating HMGB1 level in active AAV patients [15], in order to simulate the pathophysiologic conditions in AAV.

There are some limitations in this study. First, patient-derived MPO-ANCA-positive IgG rather than affinity-purity MPO-ANCA was used in most assays. The reason for this was that we were not able to acquire sufficient purified MPO-ANCA to accomplish the total experiments. However, commercial anti-MPO antibody was used; similar levels of cell activation and LDH release were observed, as compared with patient-derived MPO-ANCA-positive IgG. Second, we detected that the HMGB1 inhibition did not totally abrogate GEnC injury mediated by serum from AAV patients. The possible reason for this is that, besides HMGB1, some other proinflammatory factors in serum of AAV patients might also upregulate moesin expression. However, GEnC injury mediated by sera from AAV patients can be significantly attenuated by blocking HMGB1, suggesting that HMGB1 was important for GEnC injury.

## Conclusions

**Fig. 6:**
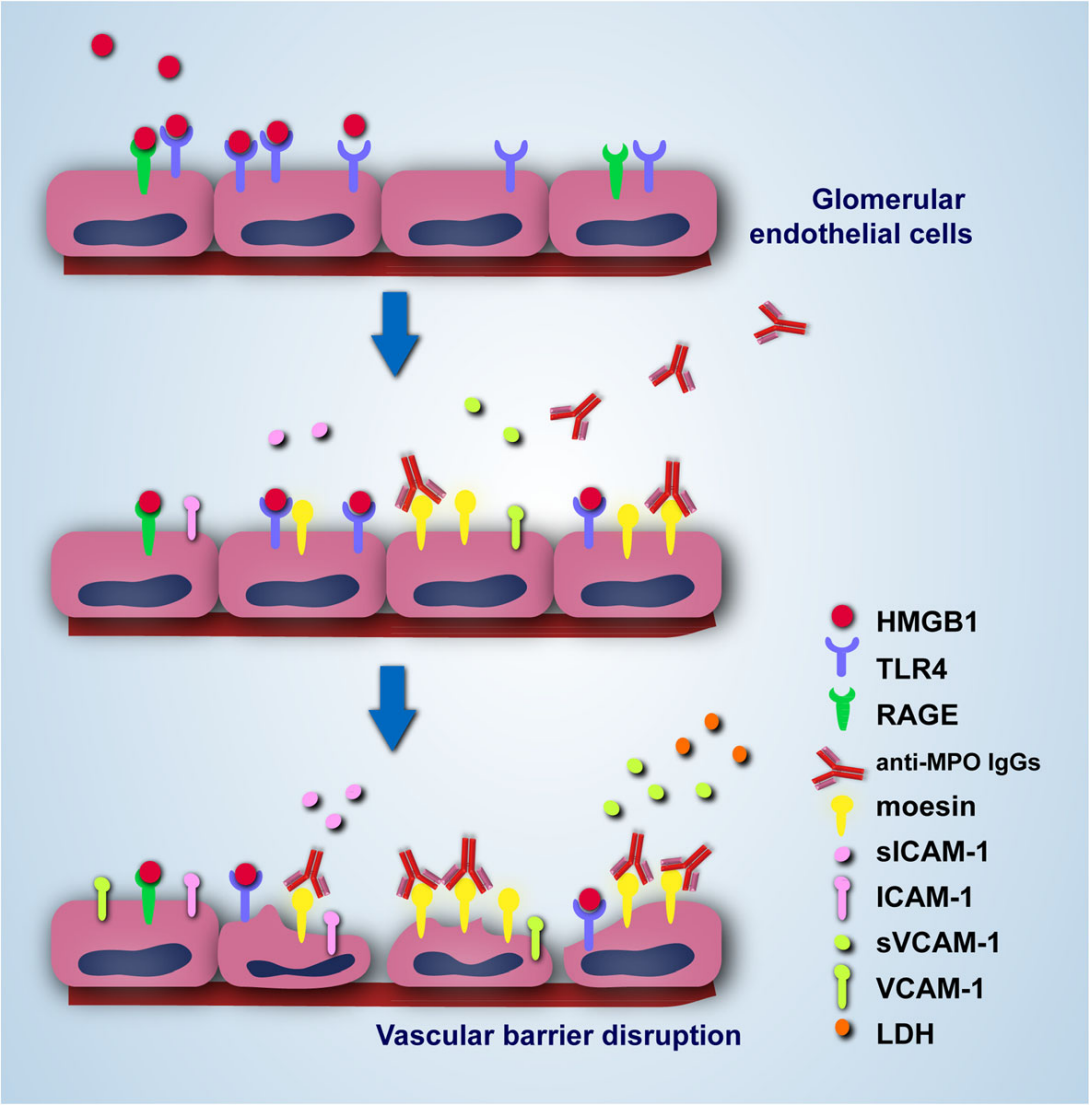
Proposed working model for the role of HMGB1 in anti-MPO antibody-induced GEnC injury. HMGB1 could increase the expression of moesin on GEnCs and further the binding of anti-MPO antibody to GEnCs due to the cross-reaction of anti-MPO antibody to moesin. In such circumstances, GEnCs could be activated and sequentially be damaged. *HMGB1* high mobility group box-1, *LDH* lactate dehydrogenase, *MPO* myeloperoxidase, *RAGE* receptor for advanced glycation end product, *sICAM*-1 soluble intercellular cell adhesion molecule-1, *sVCAM*-1 soluble vascular cell adhesion molecule-1, *TLR* Toll-like receptor

HMGB1 participates in MPO-ANCA-induced GEnC activation and injury through a moesin-dependent route, as schematized in Fig. 6. The current finding could explain, at least to some extent, the direct ability of MPO-ANCA to produce vessel damage, which provides us with more clues to determine the exact pathogenic role of ANCA in AAV.

## Additional files

Additional file 1: Table S1. Presenting a summary of the mass spectrometry results. (XLS 23 kb)

Additional file 2: Table S2. Presenting information for proteins detected in GEnCs. (XLS 632 kb)

## Abbreviations

AAV: ANCA-associated vasculitis
ANCA: Antineutrophil cytoplasmic antibody
BVAS: Birmingham Vasculitis Activity Score
ECM: Endothelial cell basal medium
EGPA: Eosinophilic granulomatosis with polyangiitis
ELISA: Enzyme-linked immunosorbent assay
ERM: Ezrin–radixin–moesin
GEnC: Glomerular endothelial cell
GPA: Granulomatosis with polyangiitis
HMGB1: High mobility group box-1
HUVEC: Human umbilical vein endothelial cells
LDH: Lactate dehydrogenase
LPS: Lipopolysaccharides
mAb: Monoclonal antibody
MPA: Microscopic polyangiitis
MPO: Myeloperoxidase
PBS: Phosphate buffer saline
PR3: Proteinase 3
RAGE: Receptor for advanced glycation end product
sICAM-1: Soluble intercellular cell adhesion molecule-1
sICAM-1: Soluble vascular cell adhesion molecule-1
TLR: Toll-like receptor
TNF-α: Tumor necrosis factor alpha
